# Tangeretin Mitigates Trimethylamine Oxide Induced Arterial Inflammation by Disrupting Choline–Trimethylamine Conversion through Specific Manipulation of Intestinal Microflora

**DOI:** 10.3390/molecules29061323

**Published:** 2024-03-16

**Authors:** Yu Cao, Changlong Leng, Kuan Lin, Youwei Li, Meiling Zhou, Mei Zhou, Xiji Shu, Wei Liu

**Affiliations:** 1 Hubei Key Laboratory of Cognitive and Affective Disorders, Wuhan Institute of Biomedical Sciences, School of Medicine, Jianghan University, Wuhan 430056, China; caoyu@stu.jhun.edu.cn (Y.C.); lengchanglong@jhun.edu.cn (C.L.); link@jhun.edu.cn (K.L.); 3495752051@stu.jhun.edu.cn (Y.L.); zhoumei@jhun.edu.cn (M.Z.); 2 Institute of Cerebrovascular Disease, School of Medicine, Jianghan University, Wuhan 430056, China

**Keywords:** TMA, tangeretin, CutC, vascular inflammation

## Abstract

Previous studies have revealed the microbial metabolism of dietary choline in the gut, leading to its conversion into trimethylamine (TMA). Polymethoxyflavones (PMFs), exemplified by tangeretin, have shown efficacy in mitigating choline-induced cardiovascular inflammation. However, the specific mechanism by which these compounds exert their effects, particularly in modulating the gut microbiota, remains uncertain. This investigation focused on tangeretin, a representative PMFs, to explore its influence on the gut microbiota and the choline–TMA conversion process. Experimental results showed that tangeretin treatment significantly attenuated the population of CutC–active bacteria, particularly *Clostridiaceae* and *Lactobacillus*, induced by choline chloride in rat models. This inhibition led to a decreased efficiency in choline conversion to TMA, thereby ameliorating cardiovascular inflammation resulting from prolonged choline consumption. In conclusion, tangeretin’s preventive effect against cardiovascular inflammation is intricately linked to its targeted modulation of TMA–producing bacterial activity.

## 1. Introduction

In recent years, the prevalence of metabolic syndromes, including cardiovascular disorders, obesity, and diabetes, has surged, posing a significant societal concern [1]. Research indicates that, beyond genetic factors, this surge is associated with the growing Westernization of dietary patterns. Specifically, there has been a shift toward higher consumption of meat and fats, including red meat, eggs, soybeans, and other foods rich in trimethylamine (TMA) substitute [2]. While traditional nutritional science has implicated trimethylamine (TMA) substitute-rich foods, and choline-rich soybeans in cardiovascular risk, recent publications in prestigious journals such as Nature [3], Nature Medicine [4], and the New England Journal of Medicine [5] propose that dietary choline may significantly elevate the risk of cardiovascular disease. This association is primarily attributed to the transformation of choline and its derivatives into TMA by gut bacteria during digestion and absorption. The results of a study on mice fed diets supplemented with trimethylamine species showed an increase in the cholesterol content of peritoneal macrophages. In contrast, dietary supplementation with choline analog 3,3–dimethyl–1–butanol did not result in an increase in macrophage cholesterol levels [3]. In addition, the study’s findings demonstrated that CutC/D TMA lyase inhibitors, such as fluoromethylcholine and iodomethylcholine, can reduce choline diet-enhanced platelet aggregation and thrombus formation in vivo [4].

Individuals with atherosclerosis have significantly higher TMA levels in their blood samples compared to those without the condition [4]. Notably, two dominant CutC species, *Lachnoclostridium* (*p* = 2.9 × 10^−5^) and *Clostridium* (*p* = 5.8 × 10^−4^), were found to be elevated in atherosclerotic patients compared to healthy controls. *L. saccharolyticum*, when exposed to choline as a substrate, converted it effectively to TMA, with a conversion rate of up to 98.7% [6]. This highlights the role of gut microbiota dysbiosis in TMAO formation and subsequent induction of atherosclerosis. Microbial TMA lyase activity, responsible for TMA production, further strengthens the link between gut microbiota and atherosclerosis [7,8]. Jonsson et al.’s study [7] indicated that the effect of gut microbiota on atherosclerosis is diet dependent, and single choline supplementation does not influence plaque size and aortic lesions. The gut microbiota utilizes three TMA lyase complexes, CntA/B, YeaW/X, and CutC/D, to convert carnitine, betaine, and choline into TMA, with CntA, YeaW, and CutC being lyases, and CntB, YeaX, and CutD serving as their activators. The relative abundance of CutC significantly increased in atherosclerosis patients (*p* = 0.033), suggesting its potential association with atherosclerosis development [6].

The unique polymethoxyflavones (PMFs) in citrus peel, such as nobiletin and tangeretin [9], have the ability to inhibit TMA generation in vivo. Nobiletin, for example, was shown in Yang et al.’s study [2] to mitigate choline-induced oxidative damage in the proximal aorta of experimental rats. It suppresses MAPK/ERK activity, reduces the expression of NF-κB p65 and phosphorylated NF–κB p65, thereby reducing inflammation. Nobiletin also demonstrated the ability to reduce TMAO-induced apoptosis of HUVEC cells and inhibit TMAO-induced proliferation of HUVEC cells. However, this study did not elucidate the regulatory mechanism of nobiletin on TMAO generation. Zhang et al. [10,11] discovered that nobiletin actively modulates the composition of gut microbiota composition, particularly affecting *Allobaculum* and *Roseburia*. These findings suggest that PMFs, as major active components of citrus peel, have the potential to shape the structure of the gut microbiota and inhibit the biological activity of TMA production.

Therefore, our study aims to investigate the targeted modulation of gut microbiota composition by tangeretin, another prominent PMF similar to nobiletin. Specifically, we focus on inhibiting TMA generation and preventing the biological activity of arterial tissue inflammation.

## 2. Results and Discussion

### 2.1. Tangeretin Effectively Counteracted the Negative Effects of Choline Chloride-Induced Inflammation

**Figure 1:**
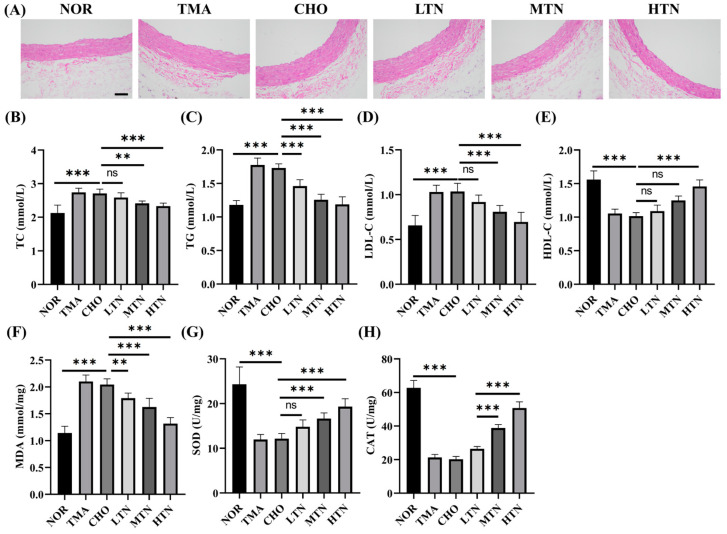
Aortic sinus HE staining results (200×, Scale bar = 200 μm) (**A**) and serum content of TC (**B**), TG (**C**), LDL–C (**D**), HDL–C (**E**), MDA (**F**), SOD (**G**), and CAT (**H**) (n = 7–8). ns: represents no significance. * Represents significant differences, ** *p* < 0.01, *** *p* < 0.001.

Histological analyses (Figure 1A) showed that choline chloride–treated rats exhibited intimal thickening, shedding of endothelial tissue, and localized elevation of the aortic intima near the heart. Additionally, elastic fibers proliferation of elastic fibers in the aorta tunica media, along with vacuolar degeneration in the intima and tunica media, indicated successful induction of cardiovascular inflammation by choline chloride. These histological manifestations are consistent with published findings indicating that TMA, derived from choline, L–carnitine, or phosphatidylcholine, can induce vascular inflammation [3,5,6]. In contrast, following tangeretin treatment, aortic intimal thickening significantly decreased, smooth muscle cells were uniformly arranged, and no smooth muscle cell hyperplasia was observed. These results collectively demonstrate that tangeretin effectively prevents choline-induced cardiovascular inflammation. Our results also indicated that the CHO group had significantly higher levels of TC (Figure 1B), TG (Figure 1C), LDL–C (Figure 1D), and a lower level of HDL–C (Figure 1E) compared to the NOR group. These changes reflected a gradual normalization of lipid metabolism and were positively correlated with histopathological alterations in the aorta. In this investigation, we assessed the protective efficacy of tangeretin on key antioxidant markers (SOD and CAT) (Figure 1G,H) and prooxidant marker (MDA) (Figure 1F), alongside its impact on pathological alterations in intestinal tissues. Notably, the CHO group showed a significant increase in MDA levels, accompanied by a substantial decrease in the enzymatic activity of SOD and CAT compared to the NOR group. This imbalance may contribute to the accumulation of reactive oxygen species (ROS), an increase in MDA concentration, and increased susceptibility to intestinal mucosa damage [12]. Conversely, tangeretin administration resulted in a significant increase in the activity of SOD and CAT enzymes, accompanied by a decrease in MDA levels. Consequently, the observed intestinal protective effect of tangeretin is likely due to its ability to mitigate lipoperoxidation, as evidenced by decreased levels of MDA levels, and the restoration of SOD and CAT levels. This restorative action is attributed to the inhibitory effect of tangeretin on oxidative damage induced by choline chloride in the colonic mucosa of experimental rats.

**Figure 2:**
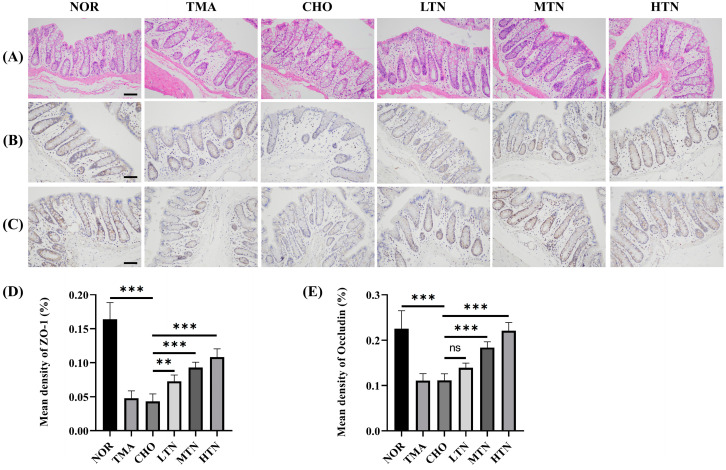
Ileal tissue HE and IHC results. (**A**) Examination of ileal tissue HE results. Immunohistochemistry (**B**) and quantification (**D**) of ZO–1. Immunohistochemistry (**C**) and quantification (**E**) of Occludin (200×). Scale bar = 200 μm, n = 7–8. ns: represents no significance. * Represents significant differences, ** *p* < 0.01, *** *p* < 0.001.

The HE staining results (Figure 2A) illustrated that choline chloride administration induced aberrant goblet cell proliferation, lamina propria tissue damage, and villous atrophy in the intestine, ultimately leading to the disruption of submucosal architecture. Pathological findings in the CHO and TMA groups suggest a direct correlation between long-term dietary intake of choline and TMA production. However, tangeretin administration significantly ameliorated these abnormal goblet cell growth patterns. IHC staining results indicated that choline chloride treatment reduced ZO-1 levels (Figure 2B,D) and Occludin levels (Figure 2C,E) in the ileum. Tangeretin treatment significantly increased the expression levels of ZO-1 (0.164 NOR, 0.048 CHO, 0.108 HTN) and Occludin (0.225 NOR, 0.111 CHO, 0.221 HTN), indicating an enhancement of intestinal tight junctions. Tight junction proteins play an essential role in stabilizing intercellular connections, preserving cell polarity, establishing specific apical domains, and controlling critical cellular activities such as proliferation, differentiation, and migration [13]. Alterations in tight junctions may be correlated with the severity of intestinal pathology [12]. In the present study, prolonged exposure to choline chloride led to increased oxidative stress levels, resulting in a deficiency in intestinal tight junctions. This impairment significantly affected the integrity of the gut epithelial barrier.

### 2.2. Tangeretin Suppressed Choline Chloride-Stimulated Inflammation

**Figure 3:**
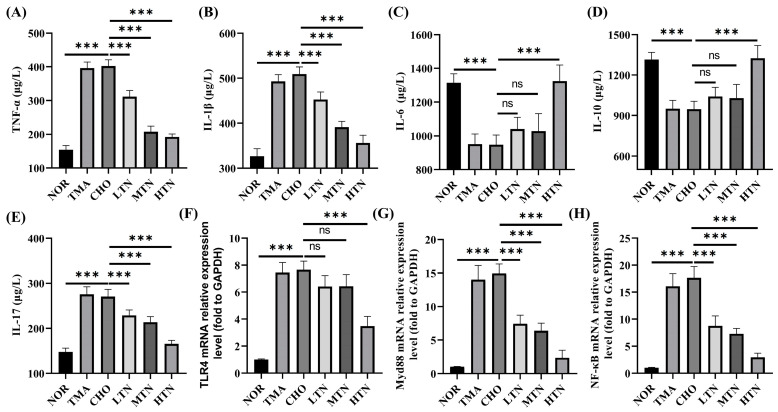
Relative expression levels of TNF-α (**A**), IL–1β (**B**), IL–6 (**C**), IL–10 (**D**), IL–17 (**E**), and aortic TLR4 (**F**), MyD88 (**G**), NF-Κb (**H**) in plasma contents (n = 7–8). ns: represents no significance. * Represents significant differences, *** *p* < 0.001.

Plasma levels of TNF–α, IL–1β, IL–6, IL–10, and IL–17, commonly used to assess inflammation severity, were significantly altered in CHO, and were reversed by the treatment of tangeretin, underscoring its potent anti-inflammatory effect (Figure 3A–E). RT–qPCR analysis revealed that choline chloride treatment significantly upregulated TLR4, MyD88, and NF-κB expression by 7.66–, 14.95–, and 17.68–fold, respectively. Conversely, concurrent administration of 200 mg/kg BW tangeretin led to a significant decrease in these transcript levels to 3.49–, 2.47–, and 2.94–fold, respectively (Figure 3F–H). The anti-inflammatory properties of tangeretin have been previously identified [14,15,16]. Therefore, our results suggest that tangeretin targets the TLR4\MyD88\NF–κB signaling pathway to inhibit the inflammatory response induced by long-term choline intake.

### 2.3. Tangeretin Regulated the Diversity of Intestinal Flora

**Figure 4:**
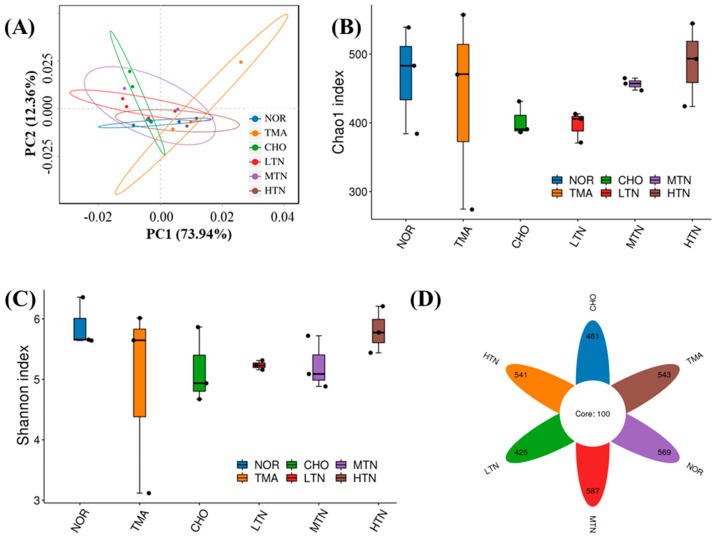
16S rDNA sequence analysis of gut microbes. (**A**) β diversity analysis of Principal Coordinate Analysis, (**B**) α diversity analysis of Chao1 index, (**C**) Shannon diversity index, and (**D**) ASV Venn diagram.

PCA analysis (Figure 4A) indicated that the NOR group was distinct from the other groups, highlighting the impact of choline chloride treatment on the composition of intestinal flora. The clustering results of the tangeretin treatment groups differed from those of the CHO group or TMA group, revealing that tangeretin treatment altered the community structure of the intestinal flora without completely reversing the changes caused by choline chloride treatment.

Choline chloride reduced both the Chao1 index, reflecting species richness, and the Shannon diversity index, indicating evenness, in the intestinal flora of rats compared to NOR. Following tangeretin treatment, the richness and evenness of the intestinal microflora exhibited a promising increase (Figure 4B,C). Fortunately, the results of Figure 4D revealed that the ASVs of the NOR, CHO, HTN, MTN, or LTN groups were 669, 561, 641, 687, and 525, respectively, suggesting alterations in the composition of the gut microbiota with the intervention of tangeretin.

### 2.4. Tangeretin Modulated the Overall Structure and Composition of Gut Microbiota

**Figure 5:**
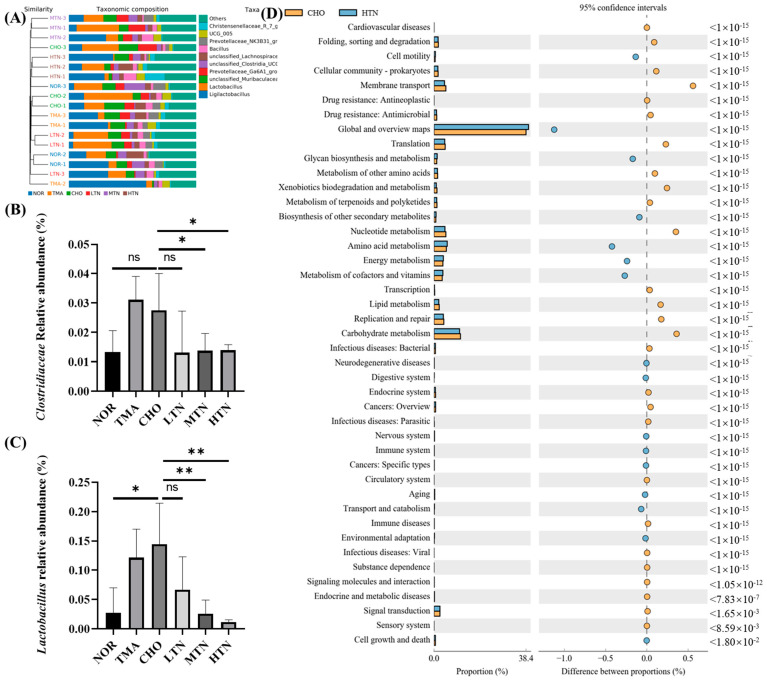
Tangeretin affected the overall pattern and composition of gut microbiota. (**A**) Gut microbiota composition at the phylum level. (**B**) Relative abundance of Clostridiaceae. (**C**) Relative abundance of Lactobacillus. (**D**) Analysis of distinctions in metabolic pathways of KEGG. Data are illustrated as means ± SD, n = 7–8. ns: represents no significance. * Represents significant differences, * *p* < 0.05, ** *p* < 0.01.

At the genus level, significant changes in abundance were observed in *Christensenellaceae*, UCG 005, *Prevotellaceae*, *Bacillus*, unclassified *Lachnospiraceae*, *Clostridia*, *Prevotellaceae*, *Murihaculaceae*, *Lactobacillus*, and *Ligilactobacillus* (Figure 5A). Notably, tangeretin treatment led to a significant downregulation of bacteria involved in choline metabolism, such as *Clostridiaceae* and *Lactobacillus* (Figure 5B,C). In our analysis of the differences in KEGG metabolic pathways between the CHO cohort and the HTN population, we observed that the differences and alterations in functional genes associated with cardiovascular disease were the most significant, with a *p*-value of <10^−15^ (Figure 5D). Previous research has also demonstrated tangeretin’s regulatory effect on the gut microbiome, promoting gut microbiota diversity and increases the population of beneficial bacteria like *Lactobacillaceae* and *Ruminococcaceae*, while concurrently decreasing the population of harmful bacteria such as *Enterococcus* and *Terrisporobacter* [17]. These findings indicated that tangeretin has the potential to affect the levels of gut commensals involved in choline metabolism into TMA, thereby mitigating valve damage from choline administration.

### 2.5. Tangeretin Inhibited the Conversion of Choline Chloride to TMA via CutC

**Figure 6:**
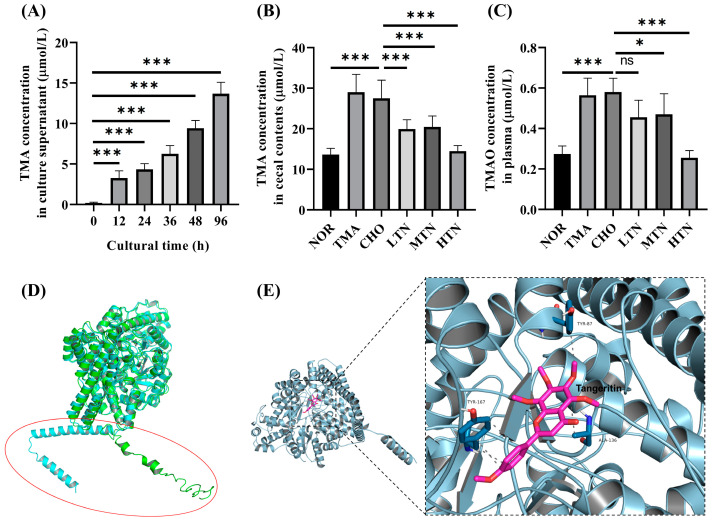
Detection of TMA and TMAO levels and molecular docking of tangeretin with CutC. (**A**) Cultural supernatant TMA level. (**B**) Cecal content TMA levels. (**C**) Plasma TMAO levels. (**D**) Computer generated structure of L. saccharolyticum WM1 CutC; (**E**) Interactions of tangeretin with important residues, n = 7–8. ns represents no significance. * Represents significant differences, * *p* < 0.05, *** *p* < 0.001.

In vitro experiments with *L. saccharolyticum* WM1 demonstrated its time-dependent conversion of choline to TMA (Figure 6A). Tangeretin significantly suppressed this conversion in a concentration-dependent inhibition manner (Figure 6B), resulting in a reduction in serum TMAO levels (Figure 6C). Published results [18,19] suggest that male rats express significantly lower levels of the FMO3 gene compared to female rats. This significant difference is clearly illustrated in Figure 6C, where even high choline/TMA administration did not cause particularly elevated TMAO levels. Based on these findings, we propose that TMA may have a proatherogenic effect, as previously reported in studies [20]. To evaluate the simulated CutC protein, ERRAT scoring was employed, revealing a value of 95.27. The PROCHECK analysis reported that 92.9% of amino acids exhibited acceptable conformations, indicating good structural integrity for the protein model. In addition, when comparing the simulated structure with the I-TASSER structure, both protein models exhibited substantial similarity in the overall tertiary structure, albeit with scattered structural transformations observed within the N-terminal residues 1–60 (Figure 6D). Subsequently, for subsequent analyses, the Robetta-modelled structure was selected for docking studies. Interaction analysis revealed that tangeretin molecules interact primarily within the hydrophobic pocket of CutC protein. The docking simulations (Figure 6E) indicated a docking energy of −32.6549 kJ/mol for the tangeretin and CutC protein complex, exceeding the threshold of −29.301 kJ/mol [21]. This result suggests a strong binding relationship between tangeretin and CutC protein. Visualization of the docking results demonstrated that tangeretin formed five conventional hydrogen bonds with amino acid residues TYR-87, ALA–136, and TYR-167 in CutC. The strong docking interaction between tangeretin and CutC provides valuable insights into the structure–function relationship between tangeretin and CutC. In conclusion, our findings confirm that long-term high–dose choline chloride treatment disrupts the gut–blood barrier, facilitating increased gut-to-blood permeation of TMA, a toxic byproduct generated by gut flora. Significantly, our study suggests that elevated plasma levels of TMAO are a consequential consequence of elevated plasma TMA, which arises from impaired intestinal barrier function in rats exposed to choline chloride.

## 3. Materials and Methods

### 3.1. Materials and Reagents

Tangeretin, with a minimum purity of 98%, was procured from Kang Biotech (Changsha, China). High-quality reagents, including TMA, 99% pure choline chloride, TMAO, and hematoxylin & eosin (HE), were sourced from Sigma–Aldrich (St. Louis, MO, USA). 3,3–Dimethylbutanol (DMB), 4% paraformaldehyde, and Oil Red O were obtained from Beyotime Biotechnology (Shanghai, China). Cholesterol package, superoxide dismutase activity colorimetric assay kit, and triglyceride package were acquired from Nanjing Jiancheng Bioengineering Institute Co., Ltd. (Nanjing, China).

### 3.2. Experimental Animal Model

Fifty Sprague–Dawley (SD) male rats aged 4–6 weeks were obtained from the Hubei Research Center of Laboratory Animals (Wuhan, China). Rats were provided unrestricted access to food and water. After one week of acclimatization, the rats were randomly assigned to six groups, each containing 7–8 animals: normal group (NOR), TMA positive group (TMA), choline model group (CHO), low tangeretin exposure group (LTN), medium tangeretin exposure group (MTN), and high tangeretin exposure group (HTN). All groups, except for NOR and TMA, were given drinking water enriched with 3% choline chloride. The exposure groups were subjected to intragastric administration dose of 50 (LTN), 100 (MTN), or 200 (HTN) mg/kg body weight (BW) of tangeretin suspended in a 1% sodium carboxymethyl cellulose aqueous solution (1 mL/dose, once daily). Additionally, the TMA group was given 200 µM/day TMA in vehicle. All experimental procedures strictly followed the ethical guidelines outlined in the European Parliament Directive on the Conservation of Animals Used for Scientific Studies (Directive 2010/63/EU) and the NIH Guide for the Care and Use of Laboratory Animals, under authorization No JHDXLL0-46 from the Experimental Animal Ethics Committee of Jianghan University.

After a 6–week period, nocturnal fasting rodents were sedated with 4% isoflurane, and blood samples were withdrawn from the left ventricle for the quantitative assessment of total cholesterol (TC), triglycerides (TG), high-density lipoprotein cholesterol (HDL–C), low-density lipoprotein cholesterol (LDL–C), aspartate transaminase (AST), and alanine transaminase (ALT) using commercial kits (Jiancheng Biotechnology Co., Ltd., Nanjing, China). Simultaneously, fecal samples were collected and stored at −80 °C for subsequent analysis, while the ileum and aortic tissues were promptly excised, immersed in cold physiological saline, or either stored at −80 °C or fixed in 4% paraformaldehyde for histopathological examination.

### 3.3. Histomorphological Staining

Specimens of the terminal ileum (proximal ileocecal valve) and proximal aortic arch were fixed in a 4% paraformaldehyde solution. Standard paraffin embedding, dewaxing, and sectioning procedures were followed, and then HE staining was performed. Pathological examination was conducted using a BH2 optical microscope (200× magnification, Olympus, Hino, Tokyo, Japan).

### 3.4. Immunohistochemical Detection

Thin sections of paraffin-embedded tissue were transferred on gelatin-coated microscope slides. After deparaffinization, rehydration, and inactivation, the sections were incubated overnight with primary antibodies (Zonula Occludens-1, ZO-1, Catalogue # 21773-1-AP or Occludin, Catalogue # 13409-1-AP from Proteintech, Wuhan, China). Following three washes with phosphate buffer, slices were exposed to HRP-labeled secondary antibody (Catalogue # ab205718, Abcam, Cambridge, USA). The specimens were stained with the chromogenic reagent 3,3′-diaminobenzidine (DAB) (Sigma-Aldrich) and counterstained with hematoxylin. The proportion of positively stained cells at each intensity level was measured using the H-score method, which assigns a score for mild staining, 2 for moderate staining, and 3 for strong staining, as described in the formula: H score = 1 × (% mild staining) + 2 × (% moderate staining) + 3 × (% strong staining).

### 3.5. RNA Isolation and Quantitative RT-qPCR

Aortic tissue was homogenized using liquid nitrogen, and total RNA was isolated with TransZol-Up reagent (TransGen Biotech Co., Ltd., Beijing, China). The quality, purity, and concentration of the RNA were assessed using a Nanodrop 2000C ultra-micro spectrophotometer (BIO-RAD T100, Hercules, USA). Reversely transcribed cDNA was synthesized from approximately 1 μg of total RNA using a HiScript™ Q RT SuperMix kit (Vazyme, Shanghai, China). Real-time quantitative polymerase chain reaction (RT-qPCR) was performed with a CFX96 real-time PCR system (ABI, Foster City, USA) for target genes such as toll-like receptor 4 (TLR4), myeloid differentiation primary response 88 (MyD88), and nuclear factor kappa-light-chain-enhancer of activated B cells (NF-κB). GAPDH served as a control. The primer sequences are delineated in Table 1, and the relative expressions of target genes were calculated using the 2^−ΔΔCt^ method.

### 3.6. Cytokine Analysis

Serum cytokine profiling, including TNF-α, IL-1β, IL-6, IL-10, and IL-17, was conducted on serum samples using ELISA (Bio-Swamp, Wuhan, China) following the provided protocol. The optical density (OD) values at a wavelength of 450 nm were measured using a microtiter plate reader (LabSystems, Helsinki, Finland).

### 3.7. Intestinal Microbiota Sequencing Procedure

The exclusive DNeasy PowerSoil Kit (Qiagen, Dusseldorf, Germany) facilitated comprehensive DNA extraction from ileal content for microbiome characterization. This involved crucial steps including DNA quantification using the sophisticated NanoDrop ND-1000 spectrophotometer (Plant & Life Science, Waltham, MA, USA), followed by electrophoresis examination via agarose gel imaging to determine purity. The bacterial taxa were amplified using fragments of the V3-V4 variable restriction fragment of the 16S RNA gene with primers 338F (5′-barcode+ACTCCTACGGGAGGCAGCA-3′) and 806R (5′-GGACTACHVGGGTWTCTAAT-3′). The purification of PCR products employed beads from Agencourt AMPure (Beckman Coulter, Brea, USA). Post-extraction, quantification was assessed using a PicoGreen dsDNA Assay Kit (Invitrogen, Carlsbad, CA, USA). Depending on the yield, multiplexed 2 × 300 bp sequencing was carried out on an Illumina MiSeq instrument using the latest MiSeq Reagent Kit v3, provided by Wuhan Servicebio Technology Co., Ltd. (Wuhan, China).

For quality control, the Quantitative Insights into Microbial Ecology pipeline (version 1.8.0) was applied to managed raw FastQ files [22]. Clustering sequence information at 97% sequence identity via UCLUST formed the basis for Operational Taxonomic Units (OTUs) [23]. These OTUs, after being aligned to the respective barcode sequences, resulted in relative abundance ASVs (Feature) data. Subsequent steps included α diversity analysis (Chao1 index and Shannon diversity index) and β diversity analysis (PCAs) [24]. The visual representation of these analyses was achieved through principal coordinate analysis (PCoA) using MEGAN [25] and GraPhlAn [26], proprietary tools from BioGraph AG, for visualizing taxonomic compositions and abundances. The R software package (version 4.2.3) Venn Diagram was employed for the visualization of shared and unique OTUs in sample or group comparisons employed the R software package Venn Diagram [27].

### 3.8. Bacterial Species and Habitats

To assess the in vitro efficiency of TMA-proliferating bacteria, *L. saccharolyticum* WM1 (ATCC 35040) was cultured under microaerophilic conditions in ATCC medium 1118 at 37 °C, as described in references [28,29]. The influence of tangeretin on choline transamination into TMA during *L. saccharolyticum* WM1 cultivation was analyzed by inoculating microbial populations with choline chloride in sterile vessels. These populations were incubated under microaerobic conditions (composed of 80% N_2_ and 20% CO_2_) at 37 °C until the cells reached an optical density at 600 nm (OD600) close to unity. Subsequently, the culture was centrifuged at approximately 15,294× *g* for 10 min at 4 °C.

### 3.9. Molecular Docking

The amino acid sequence of CutC protein from *L. saccharolyticum* WM1 was extracted from prior scientific literature [6]. Protein topological modeling was pursued using Robetta (https://robetta.bakerlab.org/submit.php (accessed on 25 November 2023)), and the structural was refined of the protein was refined using the online resources of I-TASSER (https://zhanglab.ccmb.med.umich.edu/I-TASSER/ (accessed on 29 November 2023)) online resources. After modeling, the protein constructs were optimized by using Discovery Studio 2.5 software. The protein models were reassessed using the SAVES v6.0 platform (https://saves.mbi.ucla.edu/ (accessed on 7 December 2023 )). Structural data for tangeretin were extracted from the PubChem database (https://pubchem.ncbi.nlm.nih.gov/ (accessed on 22 November 2023)) and subsequently structurally refined. Computational docking was carried out using the Autodock Vina 1.2.5, and the results were depicted using PyMOL software. Finally, pictorial renderings were formulated using PyMOL (Schrödinger, LLC. (New York, NY, USA). The PyMOL molecular graphics system, version 1.8, 2015.).

### 3.10. Measurement Quantification of TMA and TMAO

TMA in caecal contents and culture supernatant, and TMAO in plasma, were quantified using HPLC-MS/MS. Specifically, 200 mg of ileum content was precisely measured. Then, it was stirred for 10 min using a solution of acetonitrile: methanol: water (V:V:V = 40:40:20), with 2.0 μm of D9-TMA incorporated into cecal samples as an internal standard. This mixture was agitated, equilibrated at −80 °C for 2 h, and subsequently centrifugated at 15,294× *g* for 15 min at 4 °C. The supernatant was extracted and filtered through a 0.22 μm membrane for TMA evaluation [30].

For plasma samples, 20 μL of the sample was treated with 80 μL of acetonitrile for protein precipitation. Next, 2.0 μm of D9-TMAO was incorporated into plasma samples as an internal standard. The formulated mixture was agitated, left static at −80 °C for 2 h, and then centrifuged at 15,294× *g* for 15 min at 4 °C. The supernatant was extracted and filtered through a 0.22 μm membrane for TMAO determination [30].

HPLC-MS/MS analysis was performed on a TSQ Quantum Triple Quadrupole Mass Spectrometer (Thermo Fisher Scientific, Waltham, MA, USA) using a Welch Ultimate XB-C8 column (150 × 4.6 mm, 5 μm, Welch Materials, Inc., Shanghai, China) for separation. The injector temperature was 350 °C, with an injection volume of 2.00 µL. High-purity nitrogen (99.999%) was utilized as the carrier gas at a flow rate of 0.8 mL/min. The mobile phase A consisted of 0.1 mM ammonium formate (pH = 3.5), and the mobile phase B was acetonitrile. The elution conditions were as follows: 0-0.15 min, 5% of phase A and 95% of phase B; 0.15–1.2 min, 15% of phase A and 85% of phase B; 1.2–3.0 min, 20% of phase A and 80% of phase B; 3.0–6.0 min, 30% of phase A and 70% of phase B; 6.0–7.0 min, 45% of phase A and 55% of phase B; 7.0–11.0 min, 45% of phase A and 55% of phase B; 11.0–12.0 min, 5% of phase A and 95% of phase B, and sustained at 5% of phase A and 95% of phase B until completion, with a total run time of 15 min.

### 3.11. Statistical Analysis

We assessed statistical differences between groups using the Wilcoxon rank sum procedure. For multi-group comparison, the Kruskal–Wallis test, supplemented by Holm-Bonferroni adjustment, was implemented. Distinctions between groups in animal experiments were examined through single-factor variance analysis using Tukey HSD. All statistical analyses were performed using the R 3.6.1 package, and values below 0.05 were considered statistically significant.

## 4. Conclusions

Both in vivo and in vitro demonstrations confirmed the protective role of tangeretin in chloride-induced cardiovascular inflammation. In this study, we demonstrated that choline chloride induces vascular inflammation by upregulating the TLR4/MyD88/NF-κB signaling pathway, and tangeretin treatment efficaciously inhibited these inflammatory biomarkers in a dose-dependent manner, thereby preventing tissue inflammation. Essentially, tangeretin treatment not only alleviated the prolonged choline-induced damage to the intestinal mucosa, but also interfered with the intestinal microbiota-mediated conversion of choline into TMA. This dual mechanism contributes to its preventive activity against cardiovascular inflammation.

## Figures and Tables

**Table 1:** Primers used in the RT-qPCR.

Gene Name	Forward Primer (5′-3′)	Reverse Primer (5′-3′)
TLR4	GAGGACTGGGTGAGAAACGA	GCAATGGCTACACCAGGAAT
MyD88	TGTGTGTTTCCTTTGGGACA	TGCCACTACCTCATGCAAAG
NF-κB	GATGCAGTTAATGCCCCACT	TGCTGCTGGTGATTCTCTTG
GAPDH	ATGACTCTACCCACGGCAAG	GATCTCGCTCCTGGAAGATG

## Data Availability

The database that supports the conclusions of this research work will be made available by the authors upon express request.
